# Patient perceptions of osteoporosis management: a qualitative pilot study by a patient advisory group

**DOI:** 10.1007/s11657-024-01486-0

**Published:** 2025-01-15

**Authors:** R. M. Javier, F. Debiais, F. Alliot-Launois, D. Poivret, P. Bosgiraud, F. Barbe, M. Bouyssié, M. Devert, F. Roux, M. P. Sacchi, S. Sirot, P. Halbout, T. Chevalley, J. Y. Reginster, B. Cortet

**Affiliations:** 1 Rheumatology University Hospital Strasbourg, GRIO Groupe de Recherche Et d’Information Sur Les Ostéoporoses, Strasbourg, France; 2 Department of Rheumatology, University-Hospital of Poitiers, Poitiers, France; 3 AFLAR Association Française de Lutte Anti-Rhumatismale, Paris, France; 4 CHU Metz Mercy, AFLAR, Metz, France; 5 IOF International Osteoporosis Foundation, Nyon, Switzerland; 6 Biochemistry Department, College of Science, King Saud University, Riyadh, Kingdom of Saudi Arabia; 7 Department of Rheumatology and ULR 4490 (MabLab), University-Hospital of Lille, Lille, France; 8 Division of Bone Diseases, Department of Medicine, Geneva University Hospitals and Faculty of Medicine, University of Geneva, Geneva, Switzerland

**Keywords:** Fracture, Osteoporosis treatment, Quality of life, Adverse events, Treatment adherence

## Abstract

**Summary:**

The management of osteoporosis even after a fracture is declining. Our pilot study in patients with osteoporosis confirms a large ignorance of the disease and major fears and uncertainties about the treatments. Complete and sustained medical information seems essential to counteract the contradictory information, which are exclusively negative.

**Purpose:**

The management of osteoporosis (OP) even after a fracture has declined over recent years despite the actions of the medical societies concerned with this disease. The objective of this pilot study was to investigate patients’ perceptions of OP, their treatment pathways, and how information is obtained.

**Methods:**

The Association Française de Lutte Anti-Rhumatismale (AFLAR) constituted an advisory group of 7 French patients (mean age 63.7 years [54–74 years]), with various stages of OP and duration of disease. A qualitative, anonymized study was conducted with an open-ended semi-structured questionnaire, with a contribution of the International Osteoporosis Foundation (IOF) and the “Groupe de Recherche et Informations sur les Ostéoporoses” (GRIO).

**Results:**

The onset of OP was often sudden, with the fracture occurring in a context of deep misunderstanding of OP by both the public and physicians. The patients have confidence in the physician, mostly a rheumatologist, who informs about OP and initiates the treatment. The main cause of non-adherence being major fears and uncertainties about efficacy and safety of treatments. Medical information is considered as insufficient, or poorly understood. Negative information has the highest impact, even if the sources seem unreliable, such as media. There is no trust in pharmaceutical companies and the ethics of physicians are in question.

**Conclusion:**

This IOF/GRIO/AFLAR Patient Advisory Group pilot study illustrates the numerous barriers to effective OP management. Complete and sustained medical information, especially at the time of diagnosis and initiation of treatment, seems essential to counteract the contradictory information, which are exclusively negative.

## Introduction

Around 340,000 people aged ≥ 50 years living at home in France are estimated to experience osteoporotic fractures each year (EPIFRACT Study) and 60,000 individuals are hospitalized due to an osteoporotic fracture [1, 2]. A retrospective cohort study in the French health care database (FRACTOS study) reported that in the 12 months following a fracture, 58,220 patients (16.7%) received a specific osteoporosis treatment, of whom 21,228 were previously treatment naïve. The 12-month refracture rate was 6.3% and the 12-month all-cause mortality was 12.8%, ranging from 5.0% for vertebral fracture to 16.6% for hip fracture [3]. The mortality rate from osteoporosis in the year following the fracture was higher than that recorded in the year following a diagnosis of breast cancer, and close to that of myocardial infarction [3, 4].

In 2010, the French National Health Insurance Fund (CNAM) estimated the cost of fragility fractures to €4.8 billion (direct costs €1.1 billion per year + costs linked to loss of autonomy and disability). However, three-quarters of the individuals reporting fractures were never diagnosed with osteoporosis and thus did not have the opportunity to receive appropriate antifracture care [1]. In the 2021 SCOPE study, the treatment gap in France was estimated to 79% [5].

Based on this huge treatment gap and in order to identify the strategy to be developed to improve the diagnosis and management of patients with fragility fracture, the Association Française de Lutte Anti-Rhumatismale (AFLAR) in association with the International Osteoporosis Foundation (IOF) and the Groupe de Recherche et d’Information sur les Ostéoporoses (GRIO) constituted a patient advisory group to run a pilot study. The aim of this study was to explore the representations and feelings of patients facing this disease by sharing experience on specific goals such as the following: to understand the patient’s perception; to identify management pathway and the role of formal and informal caregivers; to understand how information is obtained; and to appreciate the treatment gap and patient behavior regarding treatments.

## Patients and methods

A pilot qualitative, anonymized study was conducted with an open-ended semi-structured questionnaire (Table 1). AFLAR constituted an advisory group of representative patients from the French population willing to play this role of “Advisor.” The analysis of the qualitative data was done manually using mainly a manual thematic analysis. For the recruitment of patients, an open semi-directed questionnaire was sent (Version 1 then Version 2 on 12/30/2022) to 9 women and 1 man from different regions of France and representative of the diversity of the stages of the disease-osteoporosis. Attention was also paid to recruit patients with active professional life and wide range of age. Finally, 7 women completed the questionnaire and were included in the analysis. This final sample size was determined since no additional issues or insights were identified from additional questionnaire.

**Table 1:** Questionnaire

Items
1. **Adherence to treatment** Have you, at any time, stopped your treatment or have you, at any time, been tempted to stop this treatment? If so, could you explain to us what led to this stopping or this reflection regarding a potential stopping (doctor’s opinion, other patients’ opinions, those close to them, the pharmacist’s opinion, personal reading, information via networks social, audio-visual information, etc.)? Have you discussed continuing your treatment with your pharmacist, general practitioner or specialist? Have you talked about it with someone close to you or with other patients you know? Has anyone influenced your thinking?
2. **Adverse reactions to treatment** When prescribing your treatment, were the benefits and possible side effects clearly explained to you? If yes, by whom (general practitioner, specialist, pharmacist, etc.)? Have you talked about it with those around you? Have you heard or read, in the press or on social networks, articles, reports, testimonials which could make you think that the medications which have been prescribed to you may cause side effects of which you were not informed?
**3. Trust in pharmaceutical laboratories, the press and doctors?** If so, have you talked about it with those around you, particularly with people close to you? Do you think that we can trust the communication that is orchestrated by the pharmaceutical laboratories that market osteoporosis drugs? Are they reliable? In your opinion, are doctors or other health professionals being negatively influenced by these laboratories and could their message become biased? Is the press more credible than health professionals when it comes to talking about the side effects of medications?
4. **Impact on quality of life** Does your osteoporosis affect your daily activities in any way? Has this become more or less important since you received treatment? What are your daily activities that are most impacted by your osteoporosis? Do you feel that this is having a real negative impact on your quality of life? If you had to choose a single daily activity, currently hampered by osteoporosis, and which could be improved by treatment for this disease, which would you choose? Has this impact on the quality of life and your daily activities justified someone helping you on a regular basis? If so, is it a member of your family or someone you pay or a volunteer who assists you? Is this help important to you and, in case it disappears, would this force you to consider reducing your autonomy and perhaps changing your place of residence (smaller house or apartment, living with the family, structure hospital, EPHAD…)? Since being diagnosed with osteoporosis, have you lost a certain form of autonomy? Do you feel that your osteoporosis is now well controlled or are you worried that the situation will get worse in the years to come? Alternatively, are you confident that the medications you are receiving now will allow you to lead a life almost like the one you led before osteoporosis started?
5. **Télémédecine** Since the COVID-19 pandemic, have you changed your relationships with the health professionals who care for you (general practitioner, specialist, pharmacist, rehabilitator, etc.)? Do you tend to correspond with them using new technologies (telephone, videoconferencing, etc.)? Have you reduced or increased contact with the people who usually supervise you and help you in your daily life? Was this a problem during the pandemic and do these problems persist today?
6. **Caregivers** Since being diagnosed with osteoporosis, have you lost a certain form of autonomy? In this case, are you helped in your daily activities? If someone helps you, is it a member of your family, staff you pay, or volunteers affiliated or not to a formal structure? How many hours per week do you need to feel that you are receiving adequate help? What are the main activities for which you require help (cleaning, walking, meals, etc.)? Do you have the opportunity to discuss with these caregivers your treatment, the way in which you take it, compliance with the instructions given to you by the prescriber of this treatment, your possible wish to take breaks in taking it? Do you think the advice you were given is consistent with what your doctor explained to you when prescribing the treatment (if he did so)?

## Results

Seven female patients with a mean age of 63.7 ± 2.5 years (54–74 years) were included (Table 2). Six patients had fragility fractures (1 clavicle, 1 wrist, 1 upper end fracture of the femur, 1 ankle, 4 vertebral fractures (2 VF in 2 patients), 1 patient with 10 unspecified fractures) and one patient without fracture. Duration since diagnosis of osteoporosis was very variable, from very recent diagnosis to 15 years of evolution, and representative of the diversity of stages of osteoporosis.

**Table 2:** Patients’ characteristics

Patient	1	2	3	4	5	6	7
Age (years)	67	72	54	58,5	60	74	61
Have you had one or more fractures? And if so, how many?	Yes wrist	Yes, two vertebral fractures	Yes About 10 (not specified)	Yes Hip and two vertebrae	Yes three Rib (2 ×), ankle	Yes clavicle	No
Did you fall from your height? Or another circumstance?	Yes	Yes	Yes/Yes	Yes	Yes	Yes	-
When was your osteoporosis diagnosed? at what age?		2007 57	2017 49	2022 57	2014 51	2011 63	2020 58
How was your osteoporosis diagnosed?	DXA	After the two vertebral fractures	DXA After the first fracture in 2017	DXA (after tooth fracture)	DXA (after second rib fracture)	DXA	DXA
Are you being treated for your osteoporosis?	Not specified	Yes (ttt not specified)	Yes BPs	Yes Teriparatide	Yes Aclasta (5 yrs), Prolia	Yes BPs	Yes BPs prescribed but not taken
When was treatment offered to you?		After the two vertebral fractures	After DXA	2022, after DXA	After DXA (second rib fracture)	2017 (Suggested in 2014)	After DXA
Who suggested this treatment to you?	Rheumatologist	Rheumatologist	Rheumatologist	Rheumatologist	Rheumatologist	Rheumatologist	Rheumatologist
Was this treatment offered to you quickly after the diagnosis of osteoporosis was made?	Yes	Yes	Yes	Yes	Yes	No	Yes
Had you heard of osteoporosis and its treatments before treatment was offered to you?	Yes	Never	Yes	Yes	Yes but No knowledge of treatments	Yes	Yes
Have you taken any action with your GP, specialist or pharmacist to obtain treatment for osteoporosis before it was offered to you?	No	No	No	No	No	No	No

### Entry into the disease-osteoporosis and knowledge of osteoporosis before?

Osteoporosis was diagnosed most often after the first fracture (4/7), then after a dual-energy X-ray absorptiometry (DXA) examination (2/7) and unknown for one patient. Regarding their prior knowledge of osteoporosis, the majority (5/7) had heard, with comments of patient 5: “Knowledge of osteoporosis but associated with the advanced age of women” and of patient 4 “had her mother treated for glucocorticoid-induced osteoporosis who died at age 42.” One patient has never heard of osteoporosis.

### Initiation of treatment and other personal procedures?

In all patients, a treatment was quickly proposed by the rheumatologist. Since patient 6 had hope of less aggressive treatments, another densitometric assessment was considered within 30 months. No patient had undertaken action with their general practitioner (GP), specialist or pharmacist to obtain treatment for osteoporosis before it was offered to them. Nevertheless, after a period of thinking and a dental check-up, patient 6 “asked her rheumatologist, on the advice of her primary care physician and dentist, to avoid bisphosphonates.”

### Adherence to treatments? Reasons for the shutdown? Role of the media?

- Have you, at any time or another, stopped your treatment or have you, at any time or another, been tempted to stop this treatment?

Four out of 7 patients answered yes, and one patient did even not start the treatment: “Fear about treatment…Wish to meet people undergoing treatment or discussion group in order to better understand the disease, its management, its evolution” by patient 7. For patient 2, “Treatment was suspended by my rheumatologist following osteonecrosis of the jaw. Resumption of treatment after my jaw has healed”; for patient 3, “Personal reading, opinions of other patients, press information”; for patient 5, during the infusion of my first Aclasta injection, the nurse asked me if I have seen my dentist beforehand (no, because I did not have this information). She told me to visit him very quickly, and informed me about the risks of osteonecrosis of the jaw—I started researching online and found alarming information on the risks of bisphosphonate treatments. Consulting my dentist a few days later, she got angry, asked me why I did not consult her before, talked to me about the risks of osteonecrosis of the jaw and said that this treatment was dangerous, that it remained for life in the body, that if I needed an extraction she will refer me to a stomatologist, that she herself will no longer be responsible for this, that the dental follow-up will have to be closer, and that if I have the slightest doubt/alert, I have to consult immediately; thus, I wonder “what I did by accepting this treatment – I did not know who to turn to talk about it—I did research online, looked for articles,” and finally for patient 6, “risedronate treatment was stopped temporarily due to stomach pain in connection with the generic medication, according to the rheumatologist and replaced by a gastro-resistant form.”

- Have you discussed continuing your treatment with your pharmacist, general practitioner, or specialist?

Three patients discussed with their rheumatologist and two patients stopped their medication without discussion and one patient has not started or discussed treatment but plans to do so.

- Have you talked about it with a member of your entourage or with other patients you know? Has anyone influenced your thinking?

Three patients talked with relatives but were not influenced. For patient 6, it was “YES – this whole difficult process to put in place was necessarily considered. A medical environment of friends also made it possible to discuss it, but without an ideal solution.”

### Adverse reactions to treatment

- When prescribing your treatment, were the benefits and possible side effects clearly explained to you?

For one patient, complete information were given by the rheumatologist and two patients received no information. For the others four patients, partial or not always convincing information were mentioned. For patient 2, “Very difficult to discuss the problem of side effects with the medical profession. The benefits are mainly highlighted. In any case, despite the side effects which can be very serious, after 2 fractures I had no choice.” For patient 4, “just the fact that the injection treatment must be carried out for 18 months, but no side effects mentioned,” and for patient 7, “My primary care physician advised me to follow the prescription of the rheumatologist.”

- Have you talked about it with those around you?

Four out of 7 said yes but as mentioned by patient 7, “Yes but those around me were little aware.”

- Have you heard or read, in the press or on social networks, articles, reports, testimonials which could make you think that the medications prescribed to you can cause side effects of which you were not informed? If so, have you talked about it to those around you, particularly with people close to you?

Except for one patient, all answered yes to the first question. For example, patient 2 mentioned “Study by Lausanne University Hospital on the serious side effects of Prolia, published in the newspaper Le Monde, as well as the warning in the journal Prescrire” and patient 4 “with the stomatologist for a possible removal of a wisdom tooth: risk of osteonecrosis of the jaw due to poor healing linked to treatment with Forsteo.” For the second part of the question, most patients (4/7) said yes but as mentioned by patient 7, “Yes…I talked about it but got little feedback due to lack of knowledge.”

### Trust in pharmaceutical laboratories, the press, and doctors?

- Do you think that we can trust the communication from pharmaceutical laboratories that market osteoporosis drugs? Are they reliable?

Only one patient said “yes more or less” while the majority (6/7) expressed doubts. Patient 6 mentioned “I cannot answer this question objectively as I did not have sufficient perspective on the reliability of these drugs. Some doctors remained cautious about adverse effects. Pharmaceutical laboratories do not always follow an ethical approach, and researchers sometimes play the game of commercialization.” For patient 3, “I dared to hope that communication is reliable and always in the interest of patients…..”; patient 4, “I thought we have no choice because of the risk of fractures which disable us,” and patient 7, “I did not have a clear-cut answer…I was perplexed…”.

- Biased message from doctors?

The majority (6/7) of patients did not know. For patient 3, doctors and other health professionals must keep their free will. Their training, experience, reading, research, and patient feedback on taking a medication must be sufficient without being influenced by a pharmaceutical laboratory. For patient 5 who thought that there was no bias, “Health professionals prescribe treatments with information that they receive and respect the protocols.”

- Is the press more reliable than the health professional when it comes to talking about the side effects of medications?

The majority (4/7) answered negatively to this question and 3 patients doubted. For patient 6, “Usually it is a health professional who presents the topic. We still need to know if there is any conflict of interest with the pharmaceutical compagnies” and patient 7 “It all depends on which press…”.

### Impact on daily life and quality of life?

- Does your osteoporosis affect your daily activities in any way? Has this become more or less important since you received treatment?

Apart from the patient without fracture, the six other patients were affected with major caution and fear of disability. For patient 2, “Impossible to carry a heavy load. I sold my bike, and I now fear falls a lot”; for patient 5, “Yes since I knew that I have osteoporosis,” and for patients 6, “great caution (be careful of falls! less effort possible (housework, shopping, gardening, travel limited for fear of fractures….).” Patient 3, “ I did not notice any differences since I was treated.”

- What are your daily activities that are most impacted by your osteoporosis? Do you feel that this is having a real negative impact on your quality of life?

Apart from the patient without fracture, daily activities were impacted in all patients. Patient 3 as an example said “Sports activities reduced to nothing, domestic tasks more limited, … negative impact without a doubt, I am no longer ‘me.’”.

- If you had to choose a single daily activity, currently impacted by osteoporosis, and which could be improved by treatment for this disease, which would you choose?

The majority (6/7) chose walking and sport. Their comments were to “Regain peace of mind from the fear of fracture” for patient 5 and to “live normally without pain – be able to make some physical efforts, to remain independent – no longer be dependent on loved ones. Being able to return to normal cultural activities: shows, museums, visits, trips…..” for patient 6.

- Has this impact on the quality of life and your daily activities justified someone helping you on a regular basis? If so, is it a member of your family or someone you pay or a volunteer who assists you?

Apart from the patient without fracture, all patients would need help with relatives or home help or craftsmen, especially for housework and gardening.

- Is this help important to you and, in case it disappears, would this force you to consider reducing your autonomy and perhaps changing your place of residence (smaller house or apartment, living with the family, structure hospital, nursing homes…)?

Half of the patients answered yes. For patient 4, “This allowed me to stress less, to take time for hobbies (knitting, sewing). I am living in a two-story house where the bedrooms are upstairs. I am living with my husband, but I would leave my house if I had to be alone,” and for patient 6, “This is one of my concerns, my current home, which is too big, is not suitable. I think that in the future a single storey house should be considered.”

- Do you feel that your osteoporosis is now well controlled or are you afraid that the situation will get worse in the years to come? Alternatively, are you confident that the medications you are receiving now will allow you to lead a life almost like the one you led before osteoporosis started?

Apart from two patients who answered that they did not know, the other 5 patients answered yes. For patient 2, “I think my osteoporosis will get worse as I get older, and what solution when I have explored all the existing treatments.” For patient 4, “I fear the situation will get worse. I hoped that the treatment would slow the progression of the osteoarthritis of my left hip” and patient 5 “Not confident in treatment. Major fear that my situation will deteriorate in the near future. Not the feeling that I can lead the same life as before.”

### Télémédecine

- Since the COVID-19 pandemic, have you changed your relationships with the health professionals who care for you (general practitioner, specialist, pharmacist, etc.)? Do you tend to correspond with them using new technologies (telephone, videoconferencing, etc.)?

For most of the patients (5/6), they did not change their relationships and maintained often face-to-face consultation. The comment of patient 6 was “No, not really, doctors are very rarely communicating their telephone number and email address, and I think that videoconferencing cannot always meet the patient’s expectations.” Patients 1 and 6 mentioned “difficulties in obtaining an appointment with all doctors, including the general practitioner and the dentist.” Patient 4 mentioned that physiotherapy was stopped during the first wave of COVID.

- Have you reduced or increased contact with the people who usually supervise you and help you in your daily life? Was this a problem during the pandemic and do these problems persist today?

Half of the patients answered yes since the COVID situation slowed down supervision and follow-up and the other half no.

### Caregivers

- Since being diagnosed with osteoporosis, have you lost a certain form of autonomy?

Four out of 7 answered yes, and as an example, patient 2 said “I have now to have the groceries delivered.”

- In this case, are you helped in your daily activities? If someone helps you, is it a member of your family, staff you pay, or volunteers affiliated or not to a formal structure?

Help was mainly provided by relatives including husband or partner. Patient 7 mentions that she often calls on craftsmen.

- How many hours per week do you need to feel that you are receiving adequate help?

The time of assistance granted was very variable, oscillating between 2 and 7 h per week.

- What are the main activities for which you require help (cleaning, walking, meals, etc.)?

Activities that require assistance are mainly shopping, gardening, restrictive domestic chores like vacuuming, washing floors and windows, carrying heavy objects, and anything that requires a bent over position.

- Do you have the opportunity to discuss with these caregivers your treatment, the way in which you take it, compliance with the instructions given to you by the prescriber of this treatment, your possible wish to take breaks in taking it?

Apart from patient 4 who has a caregiver who listens and respects her problems and who responds to her requests, the only two other patients who answered this question did not have an informed person with whom to address these topics.

- Do you think the advice you were given is consistent with what your doctor explained to you when prescribing the treatment (if he did so)?

Of the 3 patients who answered this question, two did not receive advice and one partially.

In summary, to alleviate misconceptions, patients express the desire to participate in discussion groups to better understand the disease, its management, and its evolution. Patients often have the feeling to receive partial or not always convincing information from the physicians, in particular on the risks and benefits linked to specific antiosteoporosis treatments. Most patients express doubts about the communication from pharmaceutical laboratories but do not think that the message from their doctors is biased, even if they would like to know if there have any conflict of interest with the pharmaceutical companies.

## Discussion

This qualitative narrative study run by a patient advisory group in patients with osteoporosis confirms and extends a large ignorance of the disease-osteoporosis and major fears and uncertainties about the treatments. Media and non-medical sources of information appear to be as or even more important for the patients than the information given by the health care providers. Information from pharmaceutical laboratories are considered as not trustful. There is agreement on a major impact on daily life (reduced activity, fear, and need for help) if fractured. Note that in France, to the contrary to some other countries, specialized osteoporosis management is mostly in rheumatologists’ hands.

These results collected from osteoporotic patients emphasize the need of improving the information to patients, health care providers, and to the general community to fight against the decrease in treatment rate in osteoporotic individuals. Whilst the difficulty to initiate a treatment in fracture-free subjects based on DXA and/or clinical risk factors is well recognized [6], particularly since the benefit of treatment is not immediately amenable, the pain, limitation in independent function after a fracture should be a strong incentive to undertake a therapy to prevent a subsequent fracture [7]. Unfortunately, many individuals who might benefit from osteoporosis treatment do not receive it since in many instances, diagnosis, evaluation, and treatment of osteoporosis, including correction of vitamin D deficiency, are not undertaken after a fragility fracture [8, 9]. Indeed, there are still barriers under these conditions, as shown in 2 interviews run in 25 patients post-fracture. These barriers, in full agreement with the results of our study, include wrong information given by health care providers such as the fracture was not caused by bone fragility, using participants appearance and X-rays to estimate bone mineral density and denying thereby the appropriateness of a DXA examination, failing to discuss fracture risk severity and giving wrong statements on treatment efficacy and safety [10]. Patients reported that they did not understand information that they received during their consultation. Therefore, it is imperative to provide patients with consistent, repeated, and accurate individualized information on risks and benefits in an accessible format to support shared decision-making. It appears that patient perception plays a central role in the decision to take a DXA examination (which is reimbursed in France), to accept the interpretation of the test, to understand the notion of fracture risk, and to initiate a pharmacological antiosteoporosis therapy [11–13]. On the other hand, general practitioners tend to underestimate the importance of osteoporosis, often considering it as aging associated process [14] or attributing the fracture to the fall, irrespective to bone fragility. Most patients have heard information regarding osteoporosis, but the link between the disease and the fracture they just experienced is often missing [15]. A systematic review and meta-synthesis of qualitative studies regarding knowledge, beliefs, and concerns about bone health has included 25 studies with a total of 757 participants (with 105 men) that has identified 4 main domains such has inadequate knowledge, beliefs and misconceptions, concerns about osteoporosis, and lack of information from health care providers [16]. The papers included were published between 2000 and 2014. Thus, the data of our study not only are in agreement with this meta-synthesis, but would unfortunately suggest that the situation has not improved in the meantime but may even worsened.

The same influences hampering antiosteoporosis treatment initiation are altering persistence and adherence to treatment [17, 18]. Key contributing factors to low adherence rates include patient’s concerns about harms, uncertainty about treatment benefits, lack of clarity about what constitutes treatment success, and insufficient follow-up. Health misinformation may even play a central role with this respect. Over the recent years, health misinformation has emerged as a prominent issue [19]. This phenomenon is related to declining trust in public authorities and in health care professionals, and spreading of uncontrolled digital media, diffusing unproved, and unreliable declaration. As reported recently, unmet need for medical care was higher among individuals who perceived a substantial degree of social media mis- and disinformation, especially among those who used social media daily and did not trust the health care system [20].

A fracture as a major risk for subsequent fracture is rarely adequately appreciated [21]. As reported in a systematic review, failure to elicit and address information needs appears to be associated with poor treatment adherence, deterioration of the doctor-patient relationship, and important psychosocial consequences [22]. Therefore, the global movement of secondary fracture prevention capture the fracture TM under the impetus of IOF aims at improving health care providers discussing strategies for fracture prevention in order to ensure that the patients understand that preventive behaviors, including drug therapy, can modify their risk [23, 24]. Since the clinical consultation is of pivotal importance in addressing barriers to treatment adherence, a recent Delphi consensus exercise has summarized patient/carer and clinician consensus regarding clearly defined tasks for clinicians in a Fracture Liaison Service model consultation [25]. These tasks included greeting/introductions; gathering information; considering therapeutic options; eliciting patient perceptions; establishing shared decision-making preferences; sharing information about osteoporosis and treatments; and checking understanding/summarizing.

This qualitative study has strengths and limitations. As strengths, the diversity of patients reflecting the heterogeneity of the disease, a questionnaire covering various aspects of the diseases, treatment, and management, and reporting using patients’ wording. As limitations should be mentioned, the low number of patients included, which precludes to estimate the relative frequency of the various beliefs and the lack of oldest old patients in the group. However, it is usual in qualitative studies, as our own study, to include a small number of patients as the inclusion of a larger number of patients does not alter the verbatim.

## Conclusion

In this IOF/GRIO/AFLAR Patient Advisory Group pilot study, numerous barriers to effective management of patients with osteoporosis were further emphasized. In addition to information for the general population, complete and sustained medical information, especially at the time of diagnosis and initiation of treatment, is of paramount importance to counteract the fake information from lay media or from insufficiently informed bodies, which are mostly negative and are likely major contributors to the large nowadays treatment gap. Sharing information through open discussions about treatment is an important step including explanation that medicine is recommended for osteoporosis or people at high risk of fracture to strengthen bones, lower the chance of future broken bones, and to maintain their functional independence. Common or severe side effects of anti-osteoporotic medication must be discussed as well as for how long the treatment is recommended. The quality of information provided to patients could also be improved through clearer informational brochures. Once the drug treatment has been discussed, it is important to check patient understanding about the benefits and risks and how the benefits are relevant for their goals. It is also important to ask about any concerns and arrange follow-up for patients who are unsure about treatment and offer helpline to discuss further.
